# Electrical Dry Needling and Therapeutic Exercise Versus Therapeutic Exercise in Patients With Chronic Nonspecific Neck Pain: Protocol for a Randomized Controlled Trial

**DOI:** 10.2196/91491

**Published:** 2026-08-20

**Authors:** Inmaculada C Lara-Palomo, Adrián Bautista-Rodríguez Sr, Jose María Muñoz-Manzaneda Sr, Elena Álvarez-López, Elena Barragán-Olea, Adelaida María Castro-Sánchez

**Affiliations:** 1 Nursing, Physical Therapy and Medicine Department, University of Almería, Road Sacramento without number, La Cañada de San Urbano, Andalusia, 04120, Spain; 2 Health Center El Ejido, Poniente de Almería Health District, Andalusian Health Service, Almeria, Spain; 3 Physical Medicine and Rehabilitation Service, Torrecárdenas University Hospital, Andalusian Health Service, Almeria, Spain

**Keywords:** chronic neck pain, therapeutic exercise, electrical dry needling, randomized controlled trial, musculoskeletal disorders, physiotherapy

## Abstract

**Background:**

Chronic nonspecific neck pain (CNSNP) is one of the most prevalent musculoskeletal disorders worldwide. Its etiology is multifactorial, and it constitutes a leading cause of work absenteeism and a significant socioeconomic burden. Therapeutic exercise, strongly supported by scientific evidence, is considered the cornerstone physiotherapy intervention for its management. In recent years, electrical dry needling (EDN) has been increasingly applied in musculoskeletal disorders with the aim of modulating pain and improving function.

**Objective:**

This protocol aims to compare the effectiveness of EDN combined with therapeutic exercise versus therapeutic exercise alone in patients with CNSNP.

**Methods:**

A randomized, controlled, examiner-blinded clinical trial with 2 parallel intervention arms will be conducted with 70 participants diagnosed with CNSNP, recruited from the outpatient physical therapy department of the University of Almería. Participants will be randomly allocated (1:1) into 2 groups: the experimental group will receive EDN combined with therapeutic exercise (n=35), while the control group will undergo a program of therapeutic exercise alone (n=35). Both groups will receive 1 session per week for 6 weeks (6 sessions in total). Primary and secondary outcome measures will include cervical disability, pain intensity, sleep quality, health-related quality of life, kinesiophobia, pain catastrophizing, cervical range of motion, pressure pain threshold at myofascial trigger points, and cervical muscle endurance. Assessments will be performed at 3 time points: baseline, postintervention, and 2-month follow-up.

**Results:**

This study was funded in 2023. Participant recruitment was conducted between November and December 2025. As of January 2026, 35 of the planned 70 participants had been recruited. Data collection is expected to be completed by March 2026. Data analysis and manuscript preparation will be carried out during 2026, with results anticipated to be published in late 2026.

**Conclusions:**

This trial will provide evidence on the effectiveness of combining EDN with therapeutic exercise in CNSNP, potentially informing clinical practice and improving patient outcomes.

## Introduction

Neck pain is the second most common musculoskeletal disorder after low back pain [1] and represents a significant public health problem [2]. It is defined as pain located between the superior nuchal line and the first thoracic vertebra, which may radiate to the head, trunk, or upper limbs [3]. Clinical manifestations include headaches, sleep disturbances, functional limitations, and negative impacts on activities of daily living, with an increased risk of falls.

Neck pain can be classified by duration: acute (<6 wk), subacute (6 wk to 3 mo), and chronic (>3 mo). Regarding etiology, it can be classified as mechanical, neuropathic, or mixed [4]. The most common form is chronic nonspecific neck pain (CNSNP), characterized by persistent pain lasting more than 3 months without an identifiable specific cause [4,5]. Common triggers include sustained postures, stress, degenerative changes, and multifactorial physical and psychosocial factors. Psychosocial factors, such as high work demands, anxiety, or depression, play a key role in the chronification of symptoms [6,7]. Due to its multifactorial nature, CNSNP poses a significant diagnostic and therapeutic challenge.

The global prevalence of neck pain ranges from 15% to 50%, with a mean of 37% [8]. Approximately half of the world’s population will experience a clinically significant episode during their lifetime, with a projected increase of 30% by 2050 due to aging and changes in work environments [8,9]. Incidence is higher in middle-aged women, particularly those with sedentary lifestyles [4,9]. In Spain, the prevalence is 9.6% in men and 22.8% in women, making it one of the leading causes of referral to physiotherapy [10]. The economic burden is substantial, including both direct costs (consultations, diagnostic tests, and medications) and indirect costs (absenteeism, disability, and lost productivity), reaching thousands of euros per patient per year in some European countries [11,12].

From a pathophysiological perspective, various hypotheses have been proposed. The “Motor Unit Rotation Recruitment Model” suggests that motor units are activated in a rotatory manner to prevent fatigue. However, factors such as stress, sustained postures, or muscle weakness can disrupt this mechanism, leading to overload, metabolic stress, nociceptive activation, and the development of myofascial trigger points (MTrPs) [10,13,14].

Clinical guidelines recommend therapeutic exercise as the first-line treatment for CNSNP in the short and medium term [13,15,16]. Therapeutic exercise has been shown to improve function, reduce pain, and provide a high cost-benefit ratio with minimal adverse effects [17-19]. Its benefits are explained by exercise-induced hypoalgesia, mediated through central and peripheral mechanisms, and by the reduction of kinesiophobia [20-22]. Commonly studied modalities include progressive cervical strengthening programs, yoga, and combined flexor-extensor exercises [17-19]. However, global exercise interventions appear to provide only short-term generalized analgesia.

In parallel, invasive techniques such as electrical dry needling (EDN) have been developed. EDN involves the insertion of a needle into MTrPs, often combined with low-frequency electrical stimulation [23,24]. This technique aims to induce a local mechanical and microtraumatic response, optimize endogenous opioid release, decrease cortisol levels, improve microcirculation, and reduce proinflammatory mediators [23,25]. These effects may contribute to improved motor control and pain reduction.

Although multiple systematic reviews support the effectiveness of therapeutic exercise in CNSNP [26,27], comparative evidence between exercise and emerging invasive techniques remains limited.

## Methods

### Study Design

The present clinical trial is justified, with the primary objective of comparing the effectiveness of EDN combined with therapeutic exercise versus therapeutic exercise alone in patients with CNSNP, evaluating its impact on disability, pain, strength, cervical range of motion, quality of life, and sleep.

This study will be conducted as a randomized, controlled, parallel-group, single-blind clinical trial (assessor-blinded), with two intervention arms: (1) EDN combined with therapeutic exercise and (2) therapeutic exercise alone in patients with CNSNP. Participants will be randomly assigned to 1 of the 2 groups in a 1:1 ratio.

This protocol has been developed in accordance with the SPIRIT (Standard Protocol Items: Recommendations for Interventional Trials) guidelines (Checklist 1). The study will be conducted at the Clinical Unit of the Faculty of Health Sciences, University of Almería, Spain. A SPIRIT flow diagram and a structured schedule of enrollment, interventions, and assessments have been included to improve methodological transparency and reproducibility.

### Ethical Considerations

This study was approved by the Research Ethics Committee of the University of Almería under reference number AP-0429‐2023-C4-F2 and was prospectively registered at ClinicalTrials.gov under identifier NCT06522893. The study will be conducted in accordance with the ethical standards of the Declaration of Helsinki (World Medical Association). All participants will receive verbal and written information about the study and will provide written informed consent before participation. The manuscript does not include an individual's data in any form (including individual images, videos, or identifiable personal details).

### Participants

A total of 70 patients aged 30 to 68 years with CNSNP of more than 3 months’ duration, who are not currently receiving other treatments, will be recruited. Participants will be recruited from internal and external populations associated with the University of Almería through flyers, emails, and informational posters. Interested individuals will contact a researcher via email or telephone, who will schedule an in-person appointment at the outpatient clinical unit affiliated with the Faculty of Health Sciences.

**Figure 1.**
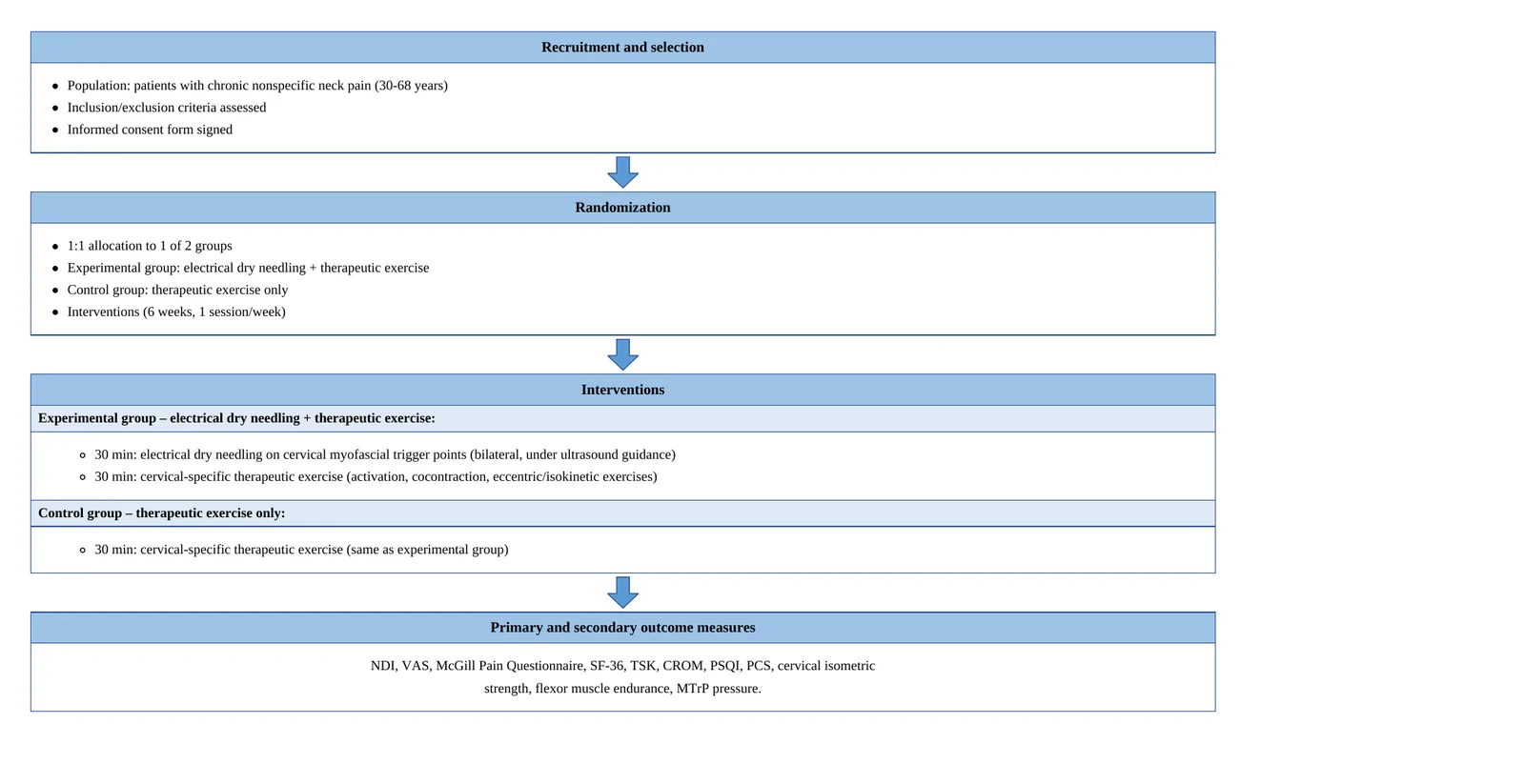
Schedule of participants and intervention characteristics. CROM: cervical range of motion; MTrPs: myofascial trigger points; NDI: Neck Disability Index; PCS: Pain Catastrophizing Scale; PSQI: Pittsburgh Sleep Quality Index; SF-36: Short Form-36 Health Survey; TSK: Tampa Scale of Kinesiophobia; VAS: Visual Analog Scale.

During this initial visit, a physiotherapist blinded to treatment allocation will assess the eligibility of participants according to the inclusion and exclusion criteria. Following this assessment, patients will be randomly assigned to 1 of 2 groups and will receive the corresponding intervention from 2 trained investigators skilled in the study techniques. All participants will provide written informed consent in accordance with the Declaration of Helsinki (World Health Organization; see Figure 1).

To improve treatment fidelity and intervention standardization, all physiotherapists involved in the study will receive prior training regarding intervention delivery, outcome assessment procedures, and adverse event reporting. Standardized treatment records and session checklists will be used throughout the study.

Participants were not directly involved in the design or conduct of this trial. However, feedback regarding feasibility, acceptability, treatment burden, and participant satisfaction will be collected during follow-up visits to inform future clinical research and implementation strategies.

To ensure participant safety and sample homogeneity, exclusion criteria were operationally defined prior to recruitment. Severe cervical pathology was defined as cervical myelopathy, progressive cervical radiculopathy with neurological deficits, fracture, tumor, inflammatory cervical disease, severe cervical stenosis, or other red-flag conditions requiring medical management. All participants underwent a standardized clinical screening based on current neck pain clinical practice guidelines before enrollment. History of spinal surgery was defined as any previous cervical spine surgical procedure, including decompression, discectomy, fusion, arthroplasty, or stabilization surgery. Coagulation disorders included diagnosed bleeding disorders or conditions requiring special precautions for needling procedures [4,28-30]. Prior to enrollment, all participants will undergo a standardized screening examination performed by a physiotherapist and confirmed by a rehabilitation physician when necessary. Screening will include medical history, neurological examination, assessment of red flags for serious spinal pathology, and review of available imaging reports when clinically indicated (Textbox 1).

Textbox 1. **Inclusion and exclusion criteria.**

**Inclusion criteria**

- Men and women aged 30-68 years.
- Chronic nonspecific neck pain lasting >3 months.
- Resting pain score ≥3 on the Numeric Pain Rating Scale.
- Not receiving other physical therapy treatment.
- Able to understand the study procedures and provide informed consent.

**Exclusion criteria**

- Clinically significant sensory disorders or coagulation disorders.
- History of spinal surgery.
- Severe cardiac disease contraindicating electrical stimulation
- Central or peripheral neurological disorders.
- Epilepsy.
- Needle phobia.
- Severe pathologies causing neck pain (eg, cervical stenosis, fracture, cervical spondylosis, tumors).
- Contraindications to transcutaneous electrical nerve stimulation.

Participants were not directly involved in the design or conduct of this trial. Feedback on the feasibility and acceptability of the intervention will be collected during follow-up to inform future research.

### Randomization, Treatment Allocation, and Adherence

Following the initial assessment, 70 patients with CNSNP will be randomly assigned to 1 of 2 groups: the experimental group receiving EDN plus specific therapeutic exercise or the control group receiving specific therapeutic exercise alone. EDN will be delivered by physiotherapists with formal postgraduate training in invasive physiotherapy and at least 3 years of clinical experience in ultrasound-guided EDN procedures.

During the study, participants will receive only the assigned intervention and will not be permitted to combine it with medications or other treatments. Any interference with the assigned treatment will result in study exclusion. Participants may withdraw from the study at any time, and the assigned interventions may be suspended or modified based on the evolution of symptoms (eg, adverse effects). All adverse events will be reported to the principal investigator, who will monitor affected patients and investigate potential causes.

To promote adherence, participants will be provided with a schedule card listing all treatment sessions, and weekly reminders will be sent via phone call, email, or WhatsApp.

### Interventions

#### Treatment Schedule and Adherence Monitoring

All participants will undergo one session per week for 6 weeks (6 sessions in total). Treatment adherence will be monitored throughout the intervention period using attendance records completed by the treating therapist. Participants will be required to attend at least 80% (5/6) of the scheduled in-person treatment sessions to be considered adherent to the protocol and eligible for inclusion in the per-protocol analysis.

#### EDN Plus Therapeutic Exercise Group

Participants allocated to the experimental group will receive 6 weekly treatment sessions of approximately 60 minutes each. Each session will consist of 30 minutes of ultrasound-guided EDN followed immediately by 30 minutes of cervical-specific therapeutic exercise.

Active MTrPs will be identified by trained physiotherapists using standardized diagnostic criteria based on current expert consensus. An active MTrP will be defined as the presence of: (1) a palpable taut band within the muscle, (2) a hypersensitive spot within the taut band, and (3) reproduction of the participant’s familiar neck pain during manual compression. When present, local twitch responses and referred pain patterns will also be recorded. Prior to participant recruitment, all treating physiotherapists will undergo calibration training sessions to standardize MTrP identification procedures [31,32].

The muscles eligible for treatment include the occipitofrontalis, sternocleidomastoid, splenius capitis, anterior, middle, and posterior scalenes, upper, middle, and lower trapezius, and multifidus at the C6 level [33]. However, only muscles demonstrating active MTrPs during clinical examination will be treated. A maximum of 6 active MTrPs will be treated per session, using 1 sterile disposable needle per trigger point.

Participants will be positioned according to the muscle being treated, and the skin will be disinfected with chlorhexidine prior to needle insertion. Sterile 0.25×25 mm acupuncture needles will be inserted under real-time ultrasound guidance to ensure accurate needle placement and patient safety. Needle depth will be individualized according to the target muscle and participant anthropometric characteristics.

Following needle insertion, electrical stimulation will be applied using a TensMed S82 (Enraf-Nonius). The stimulation parameters will be standardized at a frequency of 8 Hz and a pulse duration of 250 μs, delivered through asymmetrical biphasic square-wave currents for 30 minutes [34]. Electrical intensity will be progressively increased until participants report a strong but comfortable sensory response without pain or intolerance. The final intensity (mA) delivered during each session will be recorded.

To minimize treatment variability, all therapists will follow a standardized intervention manual detailing patient positioning, trigger point identification, needle placement procedures, stimulation parameters, and safety precautions [31,32,35]. A treatment record form will be completed after each session documenting the muscles treated, number of needles inserted, stimulation intensity, treatment duration, and any adverse events.

Participants will be informed about the possibility of postneedling soreness and will be instructed to apply local cryotherapy if required during the first 48 hours. A follow-up telephone call will be conducted 48 hours after each treatment session to monitor adverse events and ensure participant safety.

Immediately after EDN, participants will perform the same cervical-specific therapeutic exercise program received by the control group. Therefore, this trial is designed to evaluate the added value of incorporating EDN into an exercise-based physiotherapy program rather than isolating the specific physiological effects of EDN itself.

#### Therapeutic Exercise Group

Participants in the therapeutic exercise group will undergo a 6-week progressive exercise program targeting the cervical flexor and extensor muscles, with 1 session per week (6 sessions in total). The program is designed to enhance activation, recruitment, and endurance of the deep cervical flexors and extensors, improve muscle cocontraction and spinal stability, and promote neuromuscular control of the cervical spine [36,37].

Exercises are progressive, with each week incorporating the exercises from previous weeks, as detailed in Table 1, which specifies the sets, repetitions, contraction duration, and rest intervals. This structured progression aims to optimize muscle strength, endurance, and functional recovery, contributing to pain reduction and improved cervical function.

**Table 1.** Cervical spine specific exercise program.

Week, phase, and exercise	Description	Repetitions/sets	Benefit
Week 1, phase 1: activation of deep cervical flexors			
Cranio-cervical flexion (supine)	Supine with towel under neck. Slowly nod the head, activating the deep flexors without moving the larger muscles.	3×10, 10 s hold, 10 s rest	Activates deep cervical flexors, improves neck stability and posture
Cranio-cervical flexion (sitting)	Seated, feet on the floor, hips and knees at 90°. Perform slow head nods.	3×10, 10 s hold, 10 s rest	Strengthens deep flexors, improves postural control
Cervical side bending (sitting)	Tilt the head slowly to each side while keeping the shoulders relaxed.	3×10 per side, 10 s hold, 10 s rest	Improves lateral neck strength, reduces asymmetry
Scapular retraction + cranio-cervical nod	Seated or supine, gently squeeze the shoulder blades while nodding the head.	3×10, 10 s hold	Activates deep flexors and scapular stabilizers, improves posture
Chin tuck with resistance band	Supine or standing, band around forehead. Perform slow chin tucks against gentle resistance.	3×10, 10 s hold	Strengthens deep flexors, improves head alignment
Week 2, phase 2: isometric cocontraction			
Deep and superficial flexors (supine)	Physiotherapist resists cranio-cervical flexion.	10 repetitions × 10 s hold, 10 s rest	Improves cocontraction and neck stability
Flexors + rotators + lateral muscles (supine)	Flex the neck while therapist resists rotation and side bending.	10 repetitions per side × 10 s hold	Enhances multimuscle activation and motor control
Isometric cervical extension	Seated, push the head backward into the therapist’s hand without moving.	10×10 s hold	Strengthens deep extensors, reduces flexion dominance
Isometric side bending	Seated, push the head laterally against hand resistance.	10×10 s per side	Improves lateral neck endurance and symmetry
Week 3, phase 3: eccentric/isokinetic^a^			
Eccentric extensors (seated)	Extend the neck, then slowly flex to full flexion.	10 repetitions	Improves extensor control, strength, and mobility
Eccentric flexors (quadruped)	Flex the neck slowly from neutral, then extend fully.	10 repetitions	Improves flexor control, range of motion, and motor coordination
Isokinetic extensors (quadruped + resistance band)	Band around the head, perform cervical extension, then slowly flex.	10 repetitions	Enhances dynamic extensor strength and neck stability
Prone Y, T, W lifts	Prone on a table, lift the arms in Y, T, and W positions while keeping the neck neutral.	3×10	Strengthens scapular and cervical extensors, improves posture
Quadruped “cat-cow” with cranio-cervical focus	Move through flexion and extension while maintaining controlled cranio-cervical movement.	3×10, slow and controlled	Improves cervical and spinal mobility, promotes neuromuscular coordination between the cervical and thoracic spine, and reinforces controlled craniocervical movement

a The exercises and parameters from week 3 (phase 3: eccentric/isokinetic) were repeated during weeks 4 to 6 to complete the 6-week intervention period.

Therefore, this trial should be interpreted as a pragmatic effectiveness study evaluating the added value of EDN when incorporated into an exercise-based physiotherapy program rather than a mechanistic study designed to isolate the specific physiological effects of EDN.

### Data Collection and Outcome Measures

At the start of the study, demographic and clinical data will be collected, and all outcome measures will be assessed at 3 time points: baseline (preintervention), immediate posttreatment (1 d after the last session), and short-term follow-up (2 mo postintervention). Outcome assessments will be performed by a blinded evaluator using standardized procedures and calibrated instruments to improve measurement reliability and reproducibility.

The primary outcome is the Neck Disability Index (NDI), a 10-item questionnaire assessing activities of daily living. Each item is scored on a 0‐5 Likert scale. Total scores are converted to percentages (0%‐100%), with 0% indicating full independence and 100% indicating complete dependence [38,39].

Secondary outcome measures include the following:

- Visual Analog Scale: Used to assess pain intensity, participants will mark their pain level on a 100-mm horizontal line, with 0 mm representing “no pain” and 100 mm representing the “worst imaginable pain” [40].
- McGill Pain Questionnaire: A self-administered multidimensional measure of pain widely used across different patient populations, assessing both the quality and intensity of pain. It includes 3 components: sensory-discriminative, cognitive-evaluative, and affective-motivational [41].
- SF-36 (Short Form-36 Health Survey) Questionnaire: Used to assess quality of life, the SF-36 consists of 36 items across 8 subscales: physical functioning, role physical, bodily pain, general health, vitality, social functioning, role emotional, and mental health. Scores range from 0% to 100%, with higher scores indicating better perceived quality of life [42].
- Tampa Scale for Kinesiophobia: A 17-item questionnaire measuring fear of movement and (re)injury. It uses a 4-point Likert scale ranging from “strongly disagree” to “strongly agree” [43].
- Cervical range of motion: Measured using a digital goniometer (Pro Motion Capture, Werium). Participants will sit comfortably in a chair with feet flat on the floor, hips and knees at 90°, and buttocks supported against the backrest [44].
- Pressure algometry at MTrPs: A Wagner FPI 10 algometer with a rubber tip and dial will measure pressure applied to MTrPs in 0.5 kg increments. Evaluated muscles include the occipitofrontalis, splenius capitis, sternocleidomastoid, anterior, middle, and posterior scalenes, upper, middle, and lower trapezius, supraspinatus, infraspinatus, and multifidus at C6 [45].
- Pittsburgh Sleep Quality Index: Measures sleep quality and consists of 24 items, of which 19 are self-administered and 5 are for cohabitants. Items are grouped into 7 components: subjective sleep quality, sleep latency, sleep duration, habitual sleep efficiency, sleep disturbances, use of sleep medication, and daytime dysfunction. A 4-point Likert scale (0=“rarely,” 1=“sometimes,” 2=“often,” 3=“almost always”) is used, with items 2 and 5 reverse scored. Total scores range from 0 to 21, with higher scores indicating more severe sleep problems [45].
- Pain Catastrophizing Scale (PCS): A self-administered questionnaire assessing pain catastrophizing in clinical and nonclinical populations. Catastrophizing is defined as an exaggerated negative orientation toward noxious stimuli and influences pain perception and coping. The PCS consists of 13 items evaluating rumination, magnification, and helplessness, scored on a 5-point scale [46].
- Cervical isometric strength test: Maximum isometric strength of the neck extensors will be measured using a handheld dynamometer. Participants will sit with their feet flat on the floor, hips and knees at 90°, and neck in a neutral position. The handheld dynamometer will be held against the occiput with both hands, and participants will resist cervical extension isometrically for 3 seconds until maximum force is achieved [47].
- Cervical flexor endurance test: To assess maximal isometric strength of cervical flexors, participants will lie supine performing a double chin tuck and maintain this position. They will lift their head approximately 2.5 cm off the table and hold for as long as possible, while the evaluator measures endurance time. The test will end if the participant loses the position for more than 1 second or requests to stop due to pain or fatigue [48].

These outcome measures provide a comprehensive assessment of pain, disability, psychological factors, quality of life, and cervical muscle performance, allowing evaluation of both the immediate and short-term effects of the interventions.

### Sample Size Calculation

The sample size was calculated for the primary outcome, the NDI, based on detecting a clinically meaningful between-group difference of 7.5 points, which is consistent with previous studies on the responsiveness of the NDI. Assuming a standard deviation of 10.58 points, a 2-sided significance level of .05, and 80% statistical power, the required sample size was 32 participants per group. To account for an anticipated 10% (3/32) loss to follow-up, the final target sample size was increased to 35 participants per group, resulting in a total sample of 70 participants.

### Randomization

Participants will be randomly assigned to either the experimental group (EDN plus therapeutic exercise) or the control group (therapeutic exercise alone) using a computer-generated randomization sequence created by an independent statistician.

Allocation concealment will be ensured using sequentially numbered, opaque, sealed, tamper-evident envelopes prepared by an independent researcher not involved in participant recruitment, outcome assessment, or treatment administration. Envelopes will be opened sequentially only after completion of the baseline assessment and immediately before the first intervention session.

The outcome assessor will remain blinded to group allocation throughout the trial. Participants and treating physiotherapists cannot be blinded due to the nature of the interventions.

### Data Analysis

Data will be analyzed using SPSS (version 28.0; IBM Corp) and will follow the intention-to-treat principle, whereby all randomized participants will be analyzed in the groups to which they were originally assigned, regardless of treatment adherence or completion. Additionally, a per-protocol analysis will be conducted including participants who complete at least 80% (5/6) of the in-person treatment sessions. All analyses will be conducted by a statistician blinded to group allocation.

Descriptive statistics will be calculated for all variables. Continuous variables will be presented as mean (SD) or median (IQR), depending on the data distribution, while categorical variables will be expressed as frequencies and percentages.

Baseline comparability between groups will be assessed using independent Student *t* tests for normally distributed continuous variables, Mann-Whitney *U* tests for nonnormally distributed variables, and chi-square tests for categorical variables. Normality will be evaluated using the Kolmogorov-Smirnov test and visual inspection of histograms.

A statistically significant group × time interaction will be interpreted as evidence of differential treatment effects over time. To evaluate the effects of the intervention over time and between groups, linear mixed-effects models will be used, including group (experimental vs control), time (baseline, postintervention, and 2-mo follow-up), and the group × time interaction as fixed effects, with participants included as random effects. This approach allows appropriate handling of repeated measures and missing data.

If model assumptions are not met, appropriate nonparametric alternatives (eg, Friedman test for within-group comparisons and Mann-Whitney *U* test for between-group comparisons with adjusted post hoc analyses) will be applied. Adjustment for multiple comparisons among secondary outcomes will be performed using the Holm-Bonferroni procedure.

Missing data will be handled using multiple imputation methods under the assumption of missing at random. Sensitivity analyses will be conducted to assess the robustness of the findings. Dropouts and losses to follow-up will be reported and compared between groups. Twenty imputed datasets will be generated using multiple imputation procedures.

The primary end point will be the between-group difference in change in NDI scores from baseline to postintervention. Postintervention was defined as 1 day after completion of the sixth treatment session. Age, sex, and baseline NDI values will be explored as potential covariates if baseline imbalance is detected. Secondary outcomes will include changes in Visual Analog Scale, McGill Pain Questionnaire, quality of life SF-36, Tampa Scale of Kinesiophobia, cervical range of motion, Pittsburgh Sleep Quality Index, PCS, pressure pain threshold, cervical isometric strength, and cervical flexor endurance. Effect sizes (eg, Cohen *d*) will be calculated to assess the magnitude of differences.

Additionally, the clinical relevance of the findings will be assessed by comparing observed changes with established minimal clinically important differences. Based on previous studies in patients with neck pain, a change of approximately 7.5 points on the NDI and 1.5 points on pain intensity scales has been considered clinically meaningful and will be used to aid the interpretation of clinical relevance [49]. For other secondary outcomes, where established minimal clinically important differences are available, these will be considered to aid the interpretation of clinical significance.

### Masking (Blinding)

The study is single-blind; both the outcome assessor and the statistician performing data analysis will remain blinded to group allocation. Participants will be instructed not to disclose details of their intervention to the assessor. The success of blinding will be evaluated at the end of the trial by asking the assessor to guess each participant’s group assignment, and the results will be reported.

### Data Management

All participant data will be recorded on paper case report forms and stored in locked cabinets in the University of Almería outpatient clinic. Digital data will be entered into a password-protected SPSS software database.

Data entry will be double-checked for accuracy by the data manager. Backups of digital data will be maintained on a secure university server. Only authorized personnel (principal investigator, statistician, and outcome assessor) will have access to identifiable participant data.

Data will be retained for a minimum of 10 years following completion of the study, in accordance with university and national regulations.

### Adverse Event Monitoring and Management

An adverse event is defined as any undesirable and unintended sign, symptom, or medical condition occurring in a participant following receipt of a study intervention, regardless of whether a causal relationship with the administered treatment exists.

To date, no significant risks have been reported for any of the interventions; however, minor adverse effects—such as a temporary increase in pain or the development of bruising—may occur, which can be minimized through the physiotherapist’s anatomical knowledge and clinical experience.

All adverse events will be carefully monitored and recorded throughout the study. The physiotherapists responsible for the intervention will document the nature, intensity, duration, and potential causes of each adverse event, as well as any corrective actions taken.

If a significant adverse event is identified, the need to temporarily or permanently suspend the intervention for the affected participant will be assessed immediately, following predefined safety criteria. The ethics committee and the principal investigator will be notified of any serious adverse events in accordance with applicable regulatory guidelines.

Participants will also receive continuous information on the prevention and management of possible minor side effects and will be encouraged to report any new or unexpected symptoms immediately. These measures are intended to ensure participant safety and maintain the integrity of the study.

### Interim Analyses and Stopping Guidelines

No formal interim analysis is planned due to the short duration and minimal risk of the interventions. However, the study may be suspended for any participant in the event of serious adverse events, as determined by the principal investigator in consultation with the Research Ethics Committee.

### Safety Considerations

All patients and physiotherapists will receive specific information about the study in writing and will have a chance to discuss the procedures with a member of the study team before providing consent to participate. They will also be informed that they can withdraw from the study at any time. Participants who agree to participate in the study will sign 2 copies of the informed consent form, one that will be kept in the trial records and one for the participant.

The data collected from each patient will be stored in a closed locker in an office at the University of Almería, and only the physiotherapists evaluators will have access to that information. Subsequently, the data will be entered and saved by the statistician on a password-protected laptop to maintain confidentiality. Eligibility criteria, results, and analyses will not be modified after registration of the first participant.

### Follow-Up

The recruitment of patients started on November 1, 2025, and was completed by December 31, 2025. All follow-up data are expected to be collected by March 31, 2026. Data analysis, manuscript preparation, and submission to peer-reviewed scientific journals will be carried out during 2026 (Table 2).

**Table 2.** SPIRIT (Standard Protocol Items: Recommendations for Interventional Trials) schedule of enrollment, interventions, and assessments^a^.

Study period (time point)	Screening (before allocation)	Baseline (week 0)	Week 1 (session 1)	Week 2 (session 2)	Week 3 (session 3)	Week 4 (session 4)	Week 5 (session 5)	Week 6/postintervention (session 6)	2-mo follow-up (2 mo after intervention)
Enrollment									
Eligibility screening	✓								
Informed consent	✓								
Baseline demographic and clinical data		✓							
Allocation		✓							
Interventions									
Electrical dry needling + therapeutic exercise			✓	✓	✓	✓	✓	✓	
Therapeutic exercise only			✓	✓	✓	✓	✓	✓	
Assessments									
Neck Disability Index		✓						✓	✓
Visual Analog Scale		✓						✓	✓
McGill Pain Questionnaire		✓						✓	✓
SF-36^b^ Health Survey		✓						✓	✓
Tampa Scale for Kinesiophobia		✓						✓	✓
Cervical range of motion		✓						✓	✓
Pressure algometry at MTrPs^c^		✓						✓	✓
Pittsburgh Sleep Quality Index		✓						✓	✓
Pain Catastrophizing Scale		✓						✓	✓
Cervical isometric strength		✓						✓	✓
Cervical flexor endurance		✓						✓	✓
Adverse event monitoring			✓	✓	✓	✓	✓	✓	✓

a This table summarizes the schedule of enrollment, allocation, interventions, and assessments. Screening and informed consent occur before allocation. Baseline assessment and randomization are performed at week 0. The intervention period consists of 6 weekly sessions, and outcomes are assessed at baseline, immediately after the sixth session, and at 2-month follow-up.

b SF-36: Short Form-36 Health Survey.

c MTrPs: myofascial trigger points.

## Results

This study was funded in 2023 by the Fundación Progreso y Salud (reference AP-0429‐2023-C4-F2). Participant recruitment was conducted between November 1, 2025, and December 31, 2025. As of January 2026, 35 of the planned 70 participants had been recruited. Data collection for all outcome measures is expected to be completed by March 31, 2026. Data analysis, interpretation, and manuscript preparation will be carried out during 2026. The results of the trial are expected to be submitted for publication in late 2026.

## Discussion

### Comparison With Prior Work

Due to the increasing prevalence of musculoskeletal diseases, such as cervicalgia, and their socioeconomic impact globally [8-10], the importance of finding increasingly cost-effective and evidence-based treatment options for the approach increases. CNSNP is frequently associated with musculoskeletal disorders, including decreased strength and endurance of the deep cervical muscles, range of motion restrictions, postural disturbances, and peripheral and central sensitization [50,51].

EDN and therapeutic exercise are minimally invasive techniques used in the treatment of musculoskeletal pain, including chronic neck pain. EDN combines dry needling with electrical stimulation, seeking to enhance its analgesic and muscle relaxation effects [23,24]. Specific therapeutic exercise targeting the cervical region has been shown to be one of the most effective interventions for reducing pain intensity and functional disability in this population [13,15]. This type of exercise seeks to improve activation and motor control of the deep cervical muscles, restore the support and stabilization function of the cervical spine, and increase muscle strength and endurance, which contribute to improved load-bearing capacity and pain reduction. Furthermore, exercise promotes joint mobility, local circulation, and central nervous system modulation, generating both peripheral and central effects on pain perception [18,19,52].

Although both techniques are used in clinical practice and there are studies supporting their use, there is still no conclusive evidence directly comparing their efficacy and safety in patients with CNSNP. Comparing these 2 interventions with isolated therapeutic exercise can provide valuable information to determine which offers better results in terms of pain reduction, functional improvement, quality of life, and safety.

Therefore, this clinical trial emerges as a necessity to fill this gap in the evidence base, enabling more informed and evidence-based decision-making by health care professionals caring for patients with CNSNP. Furthermore, the results could contribute to optimizing treatment protocols and improving clinical outcomes in this population.

### Strengths, Limitations, and Contingency Plans

One of the main advantages of this protocol is that it is not intended to replace traditional rehabilitation but rather to provide complementary and/or parallel treatments in order to achieve more effective outcomes over time.

The main difficulties and limitations of the protocol include maintaining participant adherence throughout the course of the study, the impossibility of blinding both participants and physiotherapists—which could introduce bias—and the inability to control external aggravating factors of the pathology, such as high workload, elevated stress levels, among others. In addition, a sham EDN or attention-control intervention was not included in the study design. Therefore, nonspecific effects related to therapist contact time, patient expectations, or contextual factors cannot be completely excluded when interpreting the study findings. Consequently, the study will evaluate the effectiveness of adding EDN to usual exercise-based care rather than isolating the specific physiological effects of EDN itself. Accordingly, any observed between-group differences should be interpreted with caution, as they may reflect both specific intervention effects and nonspecific contextual influences.

Nevertheless, to facilitate adherence and minimize dropouts during the study, participants will receive reminders by phone call or WhatsApp regarding the date and time of each session. In addition, patients will be periodically informed of their improvements and progress, with the aim of motivating them to remain engaged in the treatment.

## Supplementary material

10.2196/91491 Checklist 1 SPIRIT checklist.

## Abbreviations

CNSNP: chronic nonspecific neck pain
EDN: electrical dry needling
MTrPs: myofascial trigger points
NDI: Neck Disability Index
PCS: Pain Catastrophizing Scale
SF-36: Short Form-36 Health Survey
SPIRIT: Standard Protocol Items: Recommendations for Interventional Trials
